# The association of standardized patient educators (ASPE) gynecological teaching associate (GTA) and male urogenital teaching associate (MUTA) standards of best practice

**DOI:** 10.1186/s41077-021-00162-4

**Published:** 2021-06-21

**Authors:** Holly Hopkins, Chelsea Weaks, Tim Webster, Melih Elcin

**Affiliations:** 1 Eastern Michigan University School of Nursing, 311 Marshall Building, Ypsilanti, MI 48197 USA; 2 Eastern Virginia Medical School Sentara Center for Simulation and Immersive Learning, 651 Colley Avenue, Norfolk, VA 23501 USA; 3 University of Manitoba Rady Faculty of Health Sciences, 250 Brodie Centre, 727 McDermot Avenue, Winnipeg, Manitoba R3E 3P5 Canada; 4 Department of Medical Education and Informatics, Hacettepe University Faculty of Medicine, Sihhiye Campus, 06100 Ankara, Turkey

**Keywords:** Gynecological teaching associate, Male urogenital teaching associate, Genitourinary training associate, Standardized patient, Professional patient, Standardized patient methodology, Physical examination instruction, Pelvic examination, Genitourinary examination, Rectal/prostate examination

## Abstract

Gynecological Teaching Associates (GTAs) and Male Urogenital Teaching Associates (MUTAs) instruct healthcare professional learners to perform accurate and respectful breast, speculum, bimanual vaginal, rectal, urogenital, and prostate examinations. During such sessions, the GTA/MUTA uses their own body to instruct while providing real-time feedback. While GTAs/MUTAs fall under the broader umbrella of Standardized Patient methodology, the specificity of their role indicates need for establishment of Standards of Best Practice (SOBP) for GTA/MUTA programs. On behalf of the Association of Standardized Patient Educators (ASPE), the Delphi process was utilized to reach international consensus identifying the Practices that comprise the ASPE GTA/MUTA SOBP. The original ASPE SOBP was used as the foundation for the iterative series of three surveys. Results were presented at the ASPE 2019 conference for additional feedback. Fifteen participants from four countries completed the Delphi process. Four of the original ASPE SOBP Domains were validated for GTA/MUTA programs: Safe Work Environment, Instructional Session Development, Training GTAs/MUTAs, and Program Management. Principles and Practices were shaped, and in some instances created, to best fit the distinct needs of GTA/MUTA programs. The ASPE GTA/MUTA SOBP apply to programs that engage GTAs/MUTAs in formative instructional sessions with learners. Programs that incorporate GTAs/MUTAs in simulation roles or in summative assessment are encouraged to reference the ASPE SOBP in conjunction with this document. The SOBP are aspirational and should be used to shape Practices within the program’s local context. The ASPE GTA/MUTA SOBP will continue to evolve as our knowledge-base and practice develop.

**Supplementary Information:**

The online version contains supplementary material available at 10.1186/s41077-021-00162-4.

The Association of Standardized Patient Educators (ASPE) published the ASPE Standards of Best Practice to identify guidelines for individuals and programs working with standardized patients (SPs). The ASPE SOBP identifies SPs as “a person trained to portray a patient in realistic and repeatable ways” [1]. Standardized patients may portray a patient, or they may be engaged in a purely instructional role. Gynecological Teaching Associates (GTAs) and Male Urogenital Teaching Associates (MUTAs), individuals that instruct learners to conduct breast, pelvic, rectal, urogenital, and prostate examinations are two examples of the application of SP methodology.

Historically, learners practiced pelvic examinations on plastic models, sex workers, anesthetized patients, and/or patients in clinic possibly alongside lectures, videos, and/or observation [2]. In 1972, the term Gynecology Teaching Associate was developed based on SP methodology [3]. This role combined the “patient” with the “instructor” and emphasized both technical skills and interpersonal skills. In 1979, the first publications were released addressing the male-bodied corollary, MUTAs [4, 5]. GTA and MUTA programs have been documented in the United States, Canada [6, 7], Australia [8], Belgium [9], Sweden [10], the Netherlands [11], New Zealand [12], The United Kingdom [13], England [14], and Turkey [15].

Terminology and application of methodology varies among institutions and locations (e.g., Professional Patient, Clinical Teaching Associate). As with broader SP methodology [16, 17], this variance creates some challenges related to GTA/MUTA methodology, and there is need to create consensus on the role and identify associated standards of best practice. Because GTA/MUTA interactions represent an application of SP methodology, it is ASPE’s responsibility to identify such standards as the global organization focused on human simulation. ASPE currently hosts the GTA/MUTA Special Interest Group (SIG) which has been actively engaged in coordinating workshops, activities, and courses supporting this methodology. The ASPE GTA/MUTA SIG was created in 2010 as the first professional organization to address the needs of GTA/MUTA programs.

Consistent with the ASPE SOBP, the ASPE GTA/MUTA SOBP “will provide a clear and definitive guide for all programs that” incorporate GTA/MUTA instructional sessions, with careful attention to making “these guidelines comprehensive and flexible enough to address the diversity of varying contexts” of GTA/MUTA programs [1]. The over-arching values of safety, quality, professionalism, accountability, and collaboration were identified within the ASPE SOBP [1] and apply to all SP Educators regardless of program type and/or context.

Because these standards are aspirational in nature, it is expected that institutions will be at varying degrees of implementation. The ASPE GTA/MUTA SOBP specifically addresses instructional sessions, and the ASPE SOBP should be referenced when GTAs/MUTAs are in the role of a SP or are otherwise incorporated into simulation activities and assessments. Following the ASPE GTA/MUTA SOBP will help [1] assure the physical and/or psychological safety of all participants in the session, [2] assure the effectiveness of the instructional session, and [3] decrease institutional legal risk.

## Terms related to GTA/MUTA methodology

*Gynecological Teaching Associate (GTA)*: “a female specifically trained to teach, assess, and provide feedback to learners about accurate pelvic, rectal and/or breast examination techniques. They also address the communication skills needed to provide a comfortable exam in a standardized manner, while using their own bodies [to instruct] in a supportive, non-threatening environment (ASPE)” [18].

*Male Urogenital Teaching Associate (MUTA)*: “a male specifically trained to teach, assess, and provide feedback to learners about accurate urogenital and rectal examination techniques. They also address the communication skills needed to provide a comfortable exam in a standardized manner, while using their bodies [to instruct] in a supportive, non-threatening environment (ASPE)” [18].

*SP Educator (SPE)*: “used to refer to those who work to develop expertise in SP methodology and are responsible for training and/or administering [GTA/MUTA instructional sessions]. Some may be trainers who exclusively work with SPs[/GTAs/MUTAs], while some may be faculty or healthcare professionals who work with SPs[/GTAs/MUTAs] as part of their clinical and/or academic roles” [1]. The roles of GTA and MUTA represent an application of SP methodology, therefore individuals that train or administer GTA/MUTA program are specialized SP educators.

*Instructional session*: a period of time during which an instructor teaches learners relevant clinical content and safe assessment techniques. It is anticipated that a learner has received at least minimal preparation prior to an instructional session and the GTA/MUTA builds their knowledge, competence, and confidence through hands-on practice with real-time feedback.

*Learner*: an individual who will be instructed by a GTA/MUTA. Learners are typically health professional students, practicing health professionals, or GTA/MUTA trainees.

*Participant*: a person actively engaged in an instructional session including the GTA/MUTA, and learner(s).

*Sensitive examinations*: physical assessment of the breast, vulva, vagina, uterus, adnexa, penis, scrotum/scrotal contents, inguinal region, rectum, and/or prostate.

*Stakeholder*: an individual impacted by GTA/MUTA sessions, which may include GTAs, MUTAs, staff, faculty, learners, and patients.

## Literature review

A scoping review was undertaken separately to identify the implementation and utilization characteristics of GTA and MUTA programs [19]. Articles that addressed specific domains of the SOBP were identified. While each program will implement the domains in a way that fits their unique context, this evidence is provided to demonstrate examples of implementation strategies. Textbooks were not included within the scoping review, but are also a valuable resource regarding implementation strategies [20, 21].

## Process

ASPE developed a subcommittee with the goal of using a modified Delphi process to reach international consensus identifying the Practices that comprise the ASPE GTA/MUTA SOBP. Subcommittee members were selected based on demonstration of leadership within the ASPE GTA/MUTA SIG or active ASPE participation with experience in a GTA/MUTA program. The Eastern Michigan University granted Exempt status, UHRSC-FY18–19-65.

### Delphi process

The Delphi Process was originally developed in the 1950’s as a method of predicting future trends by building consensus among experts. “Delphi may be characterized as a method for structuring a group communication process so that the process is effective in allowing a group of individuals, as a whole, to deal with a complex problem” [22]. Implementation of the Delphi Method typically includes structured group communication, anonymous responses, and assessment of views at multiple time stamps. There is broad variation in how the Delphi Method is utilized [23] and evidence to support various aspects continues to develop. While the rigorous process of repeated responses and assessment can result in participant attrition, the Delphi process features key advantages, particularly surmounting geographical limitations among participants and the elimination of the influence of strong personalities and personal agendas [24, 25].

#### Panelist recruitment

Subcommittee members (the authors of this publication) identified individuals with expertise in GTA/MUTA methodology throughout the world using the following resources: known ASPE members/contacts, authors who published on GTA/MUTA methodology, and GTA/MUTA programs with an online presence. Understanding that ASPE is an international organization with a large membership base from the United States, fifteen individuals from Australia, Belgium, New Zealand, Pakistan, Sweden, The Netherlands, the United Kingdom, and Canada were specifically invited to enhance international participation. Email invitations were additionally sent to the ASPE List-serve and the ASPE GTA/MUTA SIG List-serve. The invitation addressed the goals, the modified Delphi process, anticipated timeline, and inclusion criteria as listed:

- Must have at least 3 years of experience within a GTA and/or MUTA program
  - ○ Where GTAs and MUTAs instruct independently (as opposed to acting as models)
  - ○ In one or more of the following roles:
    - GTA or MUTA (instructing learners), and/or
    - GTA/MUTA educator/trainer (instructing GTAs/MUTAs), and/or
    - GTA/MUTA program administrator.
- Fluent in English (written)
- Currently affiliated with GTA/MUTA organization or network
- No conflict of interest

While the SOBP are directed at the program level (educators, trainers, and administrators), GTAs/MUTAs were included to represent their distinct and valuable perspective. Interested individuals emailed the Subcommittee to express interest and verify how they meet the inclusion criteria. To promote diversity, no more than one individual was to be confirmed to participate from each individual program. To ensure all stakeholders were represented, a balance of GTAs/MUTAs, GTA/MUTA educators/trainers, and GTA/MUTA administrators were selected.

There is no standardized number of panelists to include in a Delphi process, but rather researchers must identify the number that best fits the breadth and depth of their topic [26]. The intent was for at least fifteen individuals to complete the Delphi process. The selected panelists remained confidential throughout the survey process to reduce bias.

#### Survey completion

Using the ASPE SOBP as a framework, panelists completed three iterative rounds of surveys within Survey Monkey, Inc. [27]. The initial survey asked panelists to [1] rate each ASPE SOBP Practice as it pertains to GTA/MUTA programs on a 5-point scale and to provide rationale for their rating as they deem necessary then [2] brainstorm as many Practices as possible that are important to GTA/MUTA programs but were not addressed within the original ASPE SOBP. Pre-testing of the survey occurred during a workshop at the 2018 ASPE conference.

After the initial survey, the Subcommittee consolidated the qualitative responses into the list of Practices. Identical items were removed and revisions were made only for clarity and grammar, but not for content. The consolidated list was combined with the ASPE SOBP for the second survey. The second and third surveys included only the rating of the Practices on a 5-point scale (Not Applicable, Somewhat Important, Important, Very Important, Critically Important) and rationale for their rating.

Rationale provided by panelists was summarized and provided alongside the mean and standard deviation at the introduction to the second and third surveys [28]. Rationale that was opinion-based (eg., “this is how we do that at my particular organization”), as opposed to causal (eg., a reflection on the universal importance of bodily autonomy), was not considered when developing the summary [28, 29].

Each survey was available for 2 weeks. Reminder emails were sent to panelists 10 days after each survey was released. Only panelists that completed the prior survey round were invited to participate in the following survey round. An email was sent to individuals that dropped out to explore changes that would have encouraged participation.

#### Defining consensus

After each survey, the Subcommittee conducted descriptive statistics using SPSS [30] on each item. Consensus was defined as a mean of 3.8 (on a 5-point scale) with no participant rating an item as “Not Applicable”. There is no evidence-based definition of consensus due to variance in topics and groups undertaking the Delphi process, however a systematic review assessing the quality of Delphi Process reporting indicated that, among studies providing a definition of consensus, 75% was the median threshold used [23]. When an item reached consensus, it was removed from further surveys but retained in the list of Practices, statistics, and rationale provided between each survey. The Delphi process was to end when consensus was reached on all items or after three survey rounds.

Upon completion, the authors presented the ASPE GTA/MUTA SOBP at the ASPE 2019 Annual Conference to obtain feedback. All conference attendees were invited to participate in a workshop that included presentation of the survey results and the opportunity to provide feedback. Workshop attendees were divided into small groups to provide written feedback. Feedback was evaluated and incorporated into the final document. The ASPE Executive Committee approved the final document.

## Results

Twenty-one of the 26 interested individuals were selected for participation (Tables 1 and 2). While GTAs/MUTAs were qualified to participate, only one individual without educator/trainer or program administration experience expressed interest. Statistics and summarized feedback are available in the Online Supplementary File.

**Table 1:** Survey Completion

Interested	26
Qualified	25
Invited	21
Completed Consent	19
Completed Round 1	16
Completed Round 2	15
Completed Round 3	15

**Table 2:** Panelists

Participant	Institution	Country	Experience in Role(s)		
			GTA/MUTA	Educator/Trainer	Program Administrator
Amy Allen	Emory University	USA	x	x	x
Kristen Benson	Multiple in Chicago	USA	x	x	x
Carrie Bohnert	University of Louisville	USA			x
Richard Claflin	Clinical Practice Resources for Training and Education	USA	x	x	x
John Darrow	East Carolina University	USA	x	x	x
Cathelijne de Ruyter	Maastricht University	The Netherlands		x	x
Valerie Fulmer	University of Pittsburgh	USA		x	x
Holly Hopkins	Eastern Michigan University	USA	x	x	x
Hal Kerbes	University of Calgary	Canada	x	x	x
Scott Lynch-Giddings	Midwestern University	USA	x	x	x
Lynn McBain	University of Otago	New Zealand			x
Jenny Murphy	University of Michigan	USA	x	x	x
Chelsea Weaks	Eastern Virginia Medical School	USA	x	x	
Tim Webster	University of Winnipeg	Canada	x	x	x
Rose Zaeske	Johns Hopkins University	USA	x	x	x

## Domains

The Practices included within this document met statistical consensus for applicability to GTA/MUTA programs during the modified Delphi process; minor alterations were made to consensus-supported Practices to enhance clarity based on feedback as detailed above. Lack of inclusion of a Domain, Principle, or Practice included within the original ASPE SOBP does not negate the relevance of that item, but rather does [1] identify areas of future research and [2] highlight the need to consult the ASPE SOBP [1] when combining simulation or SP activities within a GTA/MUTA practices. Where appropriate, numbering of the Practices has been retained from the original ASPE SOBP [1] to maintain compatibility; although this results in gaps in the numbering of Practices within this document, it allows for effective comparison with the original ASPE SOBP document. Practices that have been omitted from this document will be listed in the introduction for each Domain. There are areas where Practices are similar, but were not combined to ensure the nuance was appropriately conveyed to the reader.

### Domain 1: safe work environment

Physical and psychological safety is critical in all working and learning environments, but it is especially critical when instructing learners to conduct sensitive examinations as GTAs/MUTAs do. This is an unique context for interaction with another person’s body and it may trigger a variety of responses for both the GTA/MUTA [31–34]; and/or learner [35–40]. Physical and psychological safety are essential for all participants, and ensuring the bodily autonomy of the GTA/MUTA and learner(s) is an effective way to facilitate this. Within each unique program, GTA/MUTA input is beneficial regarding all Practices, but in order to reduce risk of personal compromise to health or safety, the SP educator should define reasonable limits. “It is important for the GTAs[/MUTAs] to provide an opinion on how many exams are comfortable for them but within limits. I am concerned that monetary gains may cause some men/women to offer to do more exams than are safe or comfortable. I believe it is the SP educators’ job to put a reasonable limit on the number of exams” (Panelist).

“For the community of SP educators, there are three distinct principles related to creating a safe work environment: safe work practices, confidentiality, and respect” [1].

**Table:** 

Principle	Practice
1.1 Safe Work Practices	1.1.1 Ensure safe working conditions in the design of the activity (e.g., number of sessions; number of examinations; number of breaks; and physical, cognitive, and psychological challenges in the instructional session). 1.1.2 Anticipate and recognize potential occupational hazards, including threats to GTA/MUTA safety and bodily autonomy in the environment (e.g., allergenic substances, exposure to sharps, air quality, live defibrillators). 1.1.3 Screen GTAs/MUTAs to ensure that they are appropriate for the role (e.g., no conflict of interest, no compromising of their or their learner’s psychological or physical safety). 1.1.4 Allow GTAs/MUTAs to decline involvement in any activity or instructional session if they feel it is not appropriate or comfortable for them to participate (e.g., events with additional content, working during their menstrual cycle, traveling for events). 1.1.5 Brief GTAs/MUTAs so they are clear about the guidelines and parameters of an instructional session. 1.1.6 Provide GTAs/MUTAs with strategies to mitigate potential adverse effects of instructional sessions and prevent physical injury, psychological harm, or fatigue. 1.1.7 Inform GTAs/MUTAs and stakeholders about the criteria and processes for terminating an instructional session if they deem it harmful for themselves or a participant. 1.1.8 Create a process for debriefing with students and/or GTAs/MUTAs. 1.1.9 Monitor for and respond to GTAs/MUTAs who have experienced adverse effects from participation in an activity. 1.1.10 Provide a process for GTAs/MUTAs and stakeholders to report adverse effects from participation in a GTA/MUTA activity (e.g., documentation and action steps to resolve the situation). 1.1.11 Support GTAs/MUTAs who act in accordance with delineated program expectations if a complaint is made about them. 1.1.12 Manage stakeholder expectations of a GTA’s/MUTA’s possibilities and limitations. 1.1.13 Work with stakeholders to clearly define the expected scope of GTA/MUTA involvement in work assignments. 1.1.14 Define and provide clear limitations regarding the scope of skills to be covered in an instructional session (e.g., maximum number of exams per day, exam techniques that must be included in each session, exam techniques that may be instructed but not practiced with a GTA/MUTA). 1.1.15 Reinforce techniques to reduce infection risk to self and others related to sensitive examinations (e.g., proper handling of clean and contaminated equipment, hand hygiene, toileting). 1.1.16 Ensure acknowledgement of learners that they are aware of the nature of the instructional session prior to entering the room.
1.2 Confidentiality	1.2.1 Understand the specific principles of confidentiality that apply to all aspects of each instructional session. 1.2.2 Ensure that stakeholders understand and maintain the principles of confidentiality related to a specific instructional session. 1.2.3 Protect the privacy of the personal information of all stakeholders, including that which may be revealed within an instructional session. 1.2.4 Maintain instructor and learner confidentiality by protecting the privacy of any voice or video recording related to a GTA/MUTA instructional session.
1.3 Respect	1.3.1 Respect GTA’s/MUTA’s self-identified boundaries (e.g., modesty, limits to physical touch, impact on person). 1.3.2 Provide GTAs/MUTAs with adequate information so that they can make informed decisions about participation in work assignments. 1.3.3 Ensure that GTAs/MUTAs understand if and how they are being compensated before accepting work (e.g., may include payment for training and work time, travel expenses, food vouchers, gift cards).

### Domain 2: instructional session development

While the ASPE SOBP [1] Domain 2 focused on Case Development, GTAs/MUTAs primarily participate in instructional sessions, as opposed to portraying cases, indicating the need for a shift in terminology.

Many GTA/MUTA programs exist within broader SP programs or simulation centers which have a defined learning theory, feedback style, debriefing style, etc. To the extent possible, those interaction patterns should be extended to GTA/MUTA instructional sessions to provide clear support and intent for the sessions [41–44]. “The trainee should understand and expect when, where, and how feedback will be given. Feedback that comes unexpectedly, especially if it is negative, almost always is met by an emotional reaction impeding the processing of the information” [42]. Regardless of learning theory being utilized, the development of the materials to support an instructional session should be intentional.

As highlighted in the ASPE SOBP, the development of materials for instructional sessions is an iterative process of creation and refinement [1]. This is necessary to best serve the program and reflect the needs of all stakeholders. The majority of GTA/MUTA programs work with GTAs/MUTAs for formative instructional sessions; these standards reflect the need for a supportive environment that incorporates ongoing feedback from a skilled educator to support refinement of the skills being practiced [44]. If GTAs/MUTAs will be involved in summative evaluation and/or simulation, the ASPE SOBP [1] should be referenced for additional Practices that may be relevant: 2.2.9, 2.2.10, and 2.2.11.

**Table:** 

Principle	Practice
2.1 Preparation	2.1.1 Ensure that instructional materials align with measurable learning objectives. 2.1.2 Identify and engage relevant subject matter experts to assist in the creation of materials. 2.1.3 Ensure that instructional protocols are based on up-to-date clinical practice guidelines, are based on authentic problems, and respect the individuals involved in or discussed during an instructional session to avoid bias, or stereotyping marginalized populations. 2.1.4 Ensure that development of training materials allows sufficient time to draft, review, and edit materials prior to implementation. 2.1.5 Ensure that changes arising from piloting processes are addressed prior to implementation of the training materials.
2.2 Instructional Session Components	Ensure instructional session components include the following when appropriate: 2.2.1 Clear goals and objectives that can be addressed. 2.2.2 Goals and objectives that specify the intended level of learners. 2.2.3 Instructional design that meets the purpose. 2.2.4 Instructional design that is repeatable. 2.2.5 Information for GTAs/MUTAs (e.g., description of physical examination techniques, cues). 2.2.6 Training resources (e.g., equipment, videos, task trainers). 2.2.7 Guidelines for providing feedback to learners. 2.2.8 Briefing instructions, time frames, instructions to learners.

### Domain 3: GTA/MUTA training

Training may be accomplished in a variety of ways, depending on the program goals. Training curriculum should be designed in a manner consistent with the learning theories and norms of the broader SP program or simulation center, as appropriate. The objectives of the instructional session will determine the extent of the training necessary regarding knowledge of physical examination techniques and effective provision of feedback to facilitate learner confidence and competence.

GTAs/MUTAs that will provide feedback on physical examination techniques should receive training that includes a physical exam from a provider or other trained individual so that they can characterize the sensations and learn to guide learners to that goal [44]. GTAs/MUTAs should also learn the characteristics of their own body, and the particular findings that a learner will or should experience while examining their body. Individual programs should determine any restrictions, if necessary, regarding the anatomy and physiology requirements for GTAs/MUTAs to ensure that learning outcomes may be achieved during instructional sessions.

The second primary component of GTA/MUTA training involves effective and expected provision of feedback. Provision of summative feedback is clearer if the instructor understands the expected outcome: either the learner completed the task appropriately or did not. Provision of formative feedback, however, involves clarification of goals, engaging the learner in self-assessment and dialog, providing instruction on how to modify the performance, and facilitating “opportunities to close the gap between current and desired performance” all while supporting the learner’s self-confidence ([45], p. 205).

The majority of GTA/MUTA programs utilize GTAs/MUTAs for formative instructional sessions. If GTAs/MUTAs will be involved in summative evaluation and/or simulation, the ASPE SOBP [1] should be referenced for additional Practices that may be relevant: 3.2.8, 3.2.10, 3.3.6, 3.4.1–3.4.8.

**Table:** 

Principle	Practice
3.1 Preparation for Training	3.1.1 Review the purpose, objectives and outcomes, logistics, and instructional materials of the activity. 3.1.2 Address one’s own knowledge gaps, if any. 3.1.3 Create a training plan that is responsive to the context and format of each activity (e.g., group/peer training for standardization, video review, practice with simulation equipment). 3.1.4 Gather training resources to supplement training. 3.1.5 Gather administration documents and special instructions.
3.2 Training for Instructional Session	3.2.1 Review with GTAs/MUTAs the key objectives, responsibilities, context (e.g., formative, summative, level of learner, placement in curriculum) and format (e.g., length of encounter, type of encounter) of each activity. 3.2.2 Engage GTAs/MUTAs in discussion and practice of instructional session features (e.g., techniques, behaviors, expectations, and guidance to provide). 3.2.3 Provide GTA/MUTAs with strategies to deal with unanticipated learner questions, behaviors, and/or actions. 3.2.4 Ensure consistency and accuracy of instructional session of individual GTAs/MUTAs, and among groups of GTAs/MUTAs with the same role. 3.2.5 Ensure GTA/MUTA readiness for the instructional session through repeated practice and targeted feedback. 3.2.6 Provide periodic refresher or re-calibration training, even if the instructional session does not change. 3.2.7 Provide training on procedural skills (e.g., Pap collection) and equipment for training (e.g., task trainers) if utilized during instructional sessions. 3.2.9 Review current topics that are applicable to and/or potentially impact instructional sessions (e.g., consent, sexual violence, communication). 3.2.11 Educate regarding and reinforce techniques to reduce risk of physical and psychological harm to GTA/MUTA and learner (e.g., appropriate touch, appropriate pressure, consent, terminology, communication techniques). 3.2.12 Provide GTAs/MUTAs with the opportunity to demonstrate proficiency in examination maneuvers that they will be instructing. 3.2.13 Ensure pre-session resources are provided to learners to prepare them for the instructional sessions (e.g., institution-prepared materials, textbook reading assignments related to gynecological and urological exams).
3.3 Training for Feedback	3.3.1 Review with GTAs/MUTAs the fundamental principles of feedback as an instructional methodology to be applied to the planned activity. 3.3.2 Inform GTAs/MUTAs of the feedback objectives and level of the learners with whom they will be working. 3.3.3 Inform GTAs/MUTAs of the feedback logistics and setting (e.g., individual feedback with learner, small group feedback, written feedback, intended structure). 3.3.4 Train GTAs/MUTAs to use their observations, bodily sensations, and knowledge to provide feedback on observable, modifiable behaviors in learners. 3.3.5 Ensure GTA/MUTA readiness through repeated practice and targeted feedback. 3.3.7 Train GTAs/MUTAs to recognize and respond to a learner that is having a negative experience during the instructional session (e.g. history of violence, discomfort with anatomy), with the intention of ensuring a safe, nontraumatic learning environment.
3.5 Reflection on the Training Process	3.5.1 Reflect on one’s own training practices for future improvement (e.g., evaluation forms, debriefing, video review).

### Domain 4: program management

Many GTA/MUTA programs exist within broader SP and/or simulation programs but regardless of their institutional affiliation or location the broad program should follow standard business processes and procedures. This includes “creating financial management, business, and strategic plans” for the broad organization and (as applicable) specific programs [1]. The purpose for the GTA/MUTA instructional session should be determined, and should be consistent throughout. “[GTA/MUTA] programs provide a trained expert/educator in [GTA/MUTA] methodology, a trained cohort of [GTAs/MUTAs], a collaborative expertise in using [GTA/MUTA] methodology, and processes that administer [GTA/MUTA] services efficiently and cost effectively. Regardless of size, [GTA/MUTA] programs are responsible for quality management practices, including quality planning, quality assurance, quality control, and quality improvement (see INACSL Standard: Professional Integrity [8]). Clearly stated policies and procedures allow a [GTA/MUTA] program to demonstrate that it meets ethical, humanistic, professional, legislated, institutional, and practice standards for screening, recruiting, managing, and monitoring [GTAs/MUTAs] and other staff working with them” [1].

The ASPE SOBP [1] should be referenced for additional Practices as appropriate: 4.3.2, 4.3.3, 4.3.4, 4.4.1, and 4.6.1.

**Table:** 

Principle	Practice
4.1 Purpose	4.1.1 Articulate a mission statement for the program. 4.1.2 Develop program goals. 4.1.3 Identify measurable objectives for each goal (where applicable).
4.2 Expertise	4.2.1 Possess depth of knowledge in GTA/MUTA methodology. 4.2.2 Advocate for the integration of GTA/MUTA methodology into the curriculum where appropriate. 4.2.3 Identify when GTAs/MUTAs should be incorporated into an instructional session. 4.2.4 Collaborate with subject matter experts to design GTA/MUTA instructional sessions and materials. 4.2.5 Train GTAs/MUTAs according to the parameters of the instructional session.
4.3 Policies and Procedures	4.3.1 Develop and document policies to guide program activities. 4.3.4 Ensure policies and procedures are kept current and accessible. 4.3.5 Distribute policies and procedures to relevant stakeholders. 4.3.6 Develop and document policies that protect groups from discrimination (e.g., ability, age, race, ethnicity, skin color, national origin, religion, body habitus, sex, sexual orientation, gender identity, and/or gender presentation). 4.3.7 Develop and document policies for termination of an instructional session and/or case related to GTA/MUTA or learner concern. 4.3.8 Develop and document policies regarding various bodily processes that may impact and/or occur during instructional sessions and/or scenarios (e.g., discharge, vaginal bleeding, erection, passing gas or stool, infection). 4.3.9 Develop a policy or protocol for instruction of procedural skills during GTA/MUTA sessions (e.g., collection of samples). 4.3.10 Develop a policy or protocol for injury reporting and medical management if an injury occurs.
4.4 Records Management	4.4.2 Ensure that policies are in place for sharing and archiving the materials of instructional sessions. 4.4.3 Develop and document methods for securely storing, archiving, and destroying confidential data (e.g., GTA/MUTA records, learner data, audio/video data, consent forms, release forms).
4.5 Team Management	4.5.1 Consult with legal, financial, and human resources experts to ensure that status of GTAs/MUTAs (e.g., employee, independent contractor, volunteer) and compensation structure (if applicable) comply with institutional requirements. 4.5.2 Develop processes to identify, screen, interview, select, debrief, and maintain GTAs/MUTAs and staff. 4.5.3 Recruit and maintain a cohort of GTAs/MUTAs that is inclusive and diverse. 4.5.4 Establish policies and procedures for the psychological, physical, and environmental safety of GTAs/MUTAs, learners, staff, and faculty. 4.5.5 Advocate for ongoing professional development opportunities for all staff, including GTAs/MUTAs.
4.6 Quality Management	4.6.2 Gather feedback regularly from GTAs/MUTAs, learners, faculty, and other users regarding the quality of services provided by the program. 4.6.3 Analyze data and other feedback in a timely manner. 4.6.4 Implement changes for continuous improvement. 4.6.5 Inform stakeholders of changes made based on their feedback.

## Discussion

Overall, the Practices within the first 4 Domains that were omitted reflected components related to SP simulation as opposed to GTA/MUTA instructional sessions. This indicates that the consensus building process was effective. Only one panelist was lost to attrition once the survey process began, indicating that the surveys were not too complex or time-consuming.

Far more individuals expressed interest and were invited to participate than completed the process. Perspectives from 4 countries were ultimately represented although we aimed to have more global representation of panelists. While we can expect cultural differences reflected among GTA/MUTA programs in varying locations, a recent Scoping Review did not demonstrate consistent geographic variance in program specifics [19]. Although the Scoping Review indicates that it would not have significantly altered results, diversity should always be valued and encouraged. Future research should identify the degree of GTA/MUTA program implementation throughout the world and explore geographic and cultural variance among existing GTA/MUTA programs.

Three members of the research team (HH, TW, and CW) also completed the surveys for the Delphi process. They did not have access to participant responses prior to completing the surveys themselves and so were unable to impart bias. The protocol defined that all feedback and responses would be generalized from survey responses, so there was no opportunity for the researchers to impart their own individual bias. Other panelists did not know that these individuals were also participating as panelists.

The Practices within Domain 1, Safe Work Environment, reached consensus with ease. Panelists were very agreeable that safety is critical, however safety is infrequently addressed within the literature on GTA/MUTA programs. Further research should explore how SP educators perceive safety and how GTAs/MUTAs experience safety within an instructional session and simulations. The research supporting Domains 2 (Instructional Session Development), 3 (Training GTAs/MUTAs), and 4 (Program Management) is more robust, however the majority of publications are case reports detailing an individual program. Further research is indicated to demonstrate the components of effective instructional session development, strategies to optimize GTA/MUTA training, and analysis of how and whether GTA/MUTA program management is distinct from broader SP program management.

Per Lewis et al. [1], SP educators should seek professional development opportunities to further their expertise and reach. While statistical analysis only indicated consensus on Practice 5.3.2 within Domain 5, the original ASPE SOBP should be referenced by all SP educators for additional Practices that are relevant. Three of the studies addressing GTA/MUTA methodology address professional development (Table 3). Professional development is encouraged by the ASPE SOBP [1] and the Simulationist Code of Ethics [74], which both apply to all SP educators regardless of context. Additional research is indicated to determine methods for enhancing professionalization of the SP educator role, specifically engagement of GTA/MUTA methodology experts. Research should be conducted to determine whether individuals that administer GTA/MUTA programs and/or train GTAs/MUTAs identify as SP educators and whether there is a difference in perceived value that an organization places on these individuals.

**Table 3:** Evidence Supporting the Domains

	Country Represented	1 Safe Work Environment	2 Case Development	3 Training SPs	4 Program Management	5 Professional Development
Godkins, Duffy, Greenwood, & Stanhope, 1974 [46]	USA		x	x	x	
Johnson, Brown, Stenchever, Gabert, Poulson, & Warenski, 1975 [47]	USA			x	x	
Women’s Community Health Center, Inc., 1975 [48]	USA				x	
Hale & Shiner, 1977 [49]	USA		x	x	x	
Kretzschmar, 1978 [3]	USA	x	x	x	x	x
Livingstone & Ostrow, 1978 [6]	Canada		x	x		
Nelson, 1978 [50]	USA				x	
Behrens, Barnes, Gerber, Albanese, Matthes, Cangelosi, 1979 [4]	USA		x		x	
Gerber, Matthes, Albanese, 1979 [5]	USA				x	
Wheeler, Burke, Ling, 1981 [51]	USA		x	x	x	x
Laube, Kretzschmar, Guenther, Lessner, Guthrie, 1982 [52]	USA		x	x		
Fang, Hillard, Lindsay, Underwood, 1984 [53]	USA		x			
Beckmann, Sharf, Barzansky, Spellacy, 1986 [54]	USA		x			
Hillard & Fang, 1986 [55]	USA				x	
Beckmann, Barzansky, Sharf, Meyers, 1988 [56]	USA	x		x	x	x
Beckmann & Meyers, 1988 [31]	USA	x				
Muggah & Stateson, 1988 [57]	Canada			x		
Nieman, Kelliher, Sachdeva, Cohen, 1994 [58]	USA		x			
Sachdeva, Wolfson, Blair, Gillum, Gracely, Friedman, 1997 [59]	USA				x	
Costanza, Luckmann, Quirk, Clemow, White, Stoddard, 1999 [60]	USA		x	x		
Legro, Gnatuk, Kunselman, Cain, 1999 [61]	USA			x	x	
Hendrickx, De Winter, Wyndaele, Tonks, 2003 [62]	Belgium	x		x	x	
Carr & Carmody, 2004 [63]	Australia				x	
Coleman, Stewart, Wilson, Cantrell, O’Sullivan, Carthron, Wood, 2004 [64]	USA			x		
Hendrickx, de Winter, Wyndaele, Tjalma, Debaene, Selleslags, Mast, Buytaert, Bossaert, 2006 [9]	Belgium				x	
Siwe, Wijma, Stjernquist, Wijma, 2007 [65]	Sweden		x	x		
Boendermaker, Faber, Schultz, Weijmar, 2008 [66]	The Netherlands		x	x		
Robertson, Hegarty, O’Connor, & Gunn, 2008 [8]	Australia	x	x		x	
Bokken, Linssen, Scherpbier, van der Vleuten, Rethans, 2009 [67]	The Netherlands			x	x	
Jha, Setna, Al-Hity, Quinton, Roberts, 2010 [68]	United Kingdom		x		x	
Pradhan, Ebert, Brug, Swee, Ananth, 2010 [69]	USA				x	
Siebeck, Schwald, Frey, Röding, Stegmann, & Fischer, 2011 [70]	Germany			x		
Seago, Ketchum, Willett, 2012 [71]	USA				x	
Grankvist, Olofsson, Isaksson, 2014 [72]	Sweden		x	x		
Nikendei, Diefenbacher, Köhl-Hackert, Lauber, Huber, Herrmann-Werner, Herzog, Schultz, Jünger, Krautter, 2015 [32]	Germany		x	x	x	
McBain, Pullon, Garrett, Hoare, 2016 [12]	New Zealand		x		x	
Sörensdotter and Siwe, 2016 [35]	Sweden			x		
Janjua, Smith, Chu, Raut, Malick, Gallos, Singh, Irani, Gupta, Parle, Clark, 2017 [73]	United Kingdom		x			

Studies are presented in chronological order to demonstrate changes in focus over time

There were comments within the survey responses expressing concern about the use of gendered terminology in relation to GTA/MUTA sessions. The Association of American Medical Colleges’ [75] work towards inclusiveness and gender-neutral terminology is one step towards meeting that aim. Modifying terminology is beyond the scope of this project, but in the event that GTA/MUTA terminology does change in the future, this document will continue to apply to individuals and programs fulfilling the roles defined.

The ASPE GTA/MUTA SOBP demonstrates expert consensus regarding aspirational standards that all GTA/MUTA programs should aspire to uphold. These standards were designed to ensure safety, promote educational effectiveness, and reduce institutional risk. ASPE will periodically engage in rigorous methodology to ensure the GTA/MUTA SOBP continue to reflect expert consensus and growth of the roles of the SP educator and GTA/MUTA.

## Supplementary Information

**Additional file 1.** ASPE GTA MUTA SOBP Survey Results

## Data Availability

A summary of data generated or analyzed during this study are included in a supplementary information file. The datasets analyzed during the current study are available from the corresponding author on reasonable request.

## Abbreviations

ASPE: Association of Standardized Patient Educators
GTA: Gynecological Teaching Associate
MUTA: Male Urogenital Teaching Associate
SOBP: Standards of Best Practice
