# Supplementary material for: The association of standardized patient educators (ASPE) gynecological teaching associate (GTA) and male urogenital teaching associate (MUTA) standards of best practice
Source: Adv Simul (Lond). 2021 Jun 21;6:23. doi: 10.1186/s41077-021-00162-4 (PMC8215835; doi:10.1186/s41077-021-00162-4)
Supplement: Supplementary file 1 — Additional file 1. ASPE GTA MUTA SOBP Survey Results [file 41077_2021_162_MOESM1_ESM.pdf]

Online Supplemental File
ASPE GTA/MUTA SOBP Final Survey Results

	Not Applicable (1)	Somewhat Important (2)	Important (3)	Very Important (4)	Critically Important (5)

**Domain 1: Safe Work Environment**			
	**ROUND 1 RESULTS**	**ROUND 2 RESULTS**	**ROUND 3 RESULTS**
**1.1 Safe Work Practices**			
1.1.1. Ensure safe working conditions in the design of the activity (e.g., number of rotations, number of breaks, physical, cognitive, and psychological challenges in the role portrayal).	Mean (SD): 4.9375 (0.25)<br>Comments: "Ensure safe working conditions in the design of the activity (e.g., number of sessions, number of invasive examinations, number of breaks, physical, cognitive, and psychological challenges in the instructional session)." Consider adding number of invasive exams and bodily autonomy as part of the examples, also substituting "exam" for "rotation",	Mean (SD): 4.933 (0.25820)<br>Comments: Consensus reached in Round 2.	Consensus reached in Round 2.
1.1.2 Anticipate and recognize potential occupational hazards, including threats to GTA/MUTA safety in the environment (e.g., allergenic substances, exposure to sharps, air quality, live defibrillators).	Mean (SD): 4.6875 (0.60208)<br>Comments: "Anticipate and recognize potential occupational hazards, including threats to GTA/MUTA safety in the environment (e.g., bodily autonomy, allergenic substances, exposure to sharps, air quality, live defibrillators)." Consider including body autonomy in example	Mean (SD): 4.5333 (0.74322)<br>Comments: Consensus reached in Round 2.	Consensus reached in Round 2.

1.1.3 Screen GTAs/MUTAs to ensure that they are appropriate for the role (e.g., no conflict of interest, no compromising of their psychological or physical safety).	Mean (SD): 4.69 (0.48)<br>Comments: “Screen GTAs/MUTAs to ensure that they are appropriate for the role (e.g., no conflict of interest, no compromising of their or their learner’s psychological or physical safety).” Consider adding student’s psychological safety, consider changing “no compromising”	Mean (SD): 4.6667 (0.61721)<br>Comments: Consensus reached in Round 2.	Consensus reached in Round 2.
1.1.4. Allow GTAs/MUTAs to opt out of any given activity if they feel it is not appropriate for them to participate.	Mean (SD): 4.50 (1.10)<br>Comments: “Allow GTAs/MUTAs to opt out of any given activity if they feel it is not appropriate or comfortable for them to participate.” Based on comments reflecting comfort with the role of GTA/MUTA.	Mean (SD): 4.40000 (1.24212)<br>Comments: “Allow GTAs/MUTAs to decline involvement in any activity or instructional session if they feel it is not appropriate or comfortable for them to participate (e.g., events with additional content, working during their menstrual cycle, traveling for events).” Revisions based on comments that reflect needing to clearly define the role of the GTA/MUTA and that they cannot opt to exclude anything that is prescribed in the role.	Mean (SD): 4.73 (.46)<br>Comments: Consensus reached in Round 3
1.1.5 Brief GTAs/MUTAs so they are clear about the guidelines and parameters of a simulation activity.	Mean (SD): 4.13 (1.31)<br>Comments: “Brief GTAs/MUTAs so they are clear about the guidelines and parameters of an instructional session.” Based on comments reflecting inaccuracy of terminology.	Mean (SD): 4.7333 (0.45774)<br>Comments: Consensus reached in Round 2.	Consensus reached in Round 2.
1.1.6 Provide GTAs/MUTAs with	Mean (SD): 4.13 (1.09)	Mean (SD): 4.5333 (0.63994)	Consensus reached in Round 2.

strategies to mitigate potential adverse effects of role portrayal and prevent physical injury or fatigue.	Comments: “Provide GTAs/MUTAs with strategies to mitigate potential adverse effects of instructional sessions and prevent physical injury, psychological harm, or fatigue.” Based on comments reflecting inaccuracy of terminology and inclusion of psychological harm.	Comments: Consensus reached in Round 2.	
1.1.7 Inform GTAs/MUTAs and clients about the criteria and processes for terminating a simulation if they deem it harmful.	Mean (SD): 4.31 (1.08) Comments: “Inform GTAs/MUTAs and clients about the criteria and processes for terminating an instructional session if they deem it harmful for themselves or a participant.” Based on comments reflecting inaccuracy of terminology and inclusion of student safety.	Mean (SD): 4.667 (0.91548) Comments: Consensus reached in Round 2.	Consensus reached in Round 2.
1.1.8 Structure time and create a process for de-roling and/or debriefing.	Mean (SD): 3.44 (1.15) Comments: “Create a process for debriefing with students and/or GTAs/MUTAs.” Based on comments reflecting de-rolling being non-applicable, and debriefing as an optional but necessary process to have prepared.	Mean (SD): 4.0000 (0.75593) Comments: Consensus reached in Round 2.	Consensus reached in Round 2.
1.1.9 Monitor for and respond to GTAs/MUTAs who have experienced adverse effects from participation in an activity.	Mean (SD): 4.75 (0.45) Comments: Consensus reached in Round 1	Consensus reached in Round 1	Consensus reached in Round 1
1.1.10 Provide a process for	Mean (SD): 4.73 (0.46)	Consensus reached in Round 1	Consensus reached in Round 1

GTAs/MUTAs and clients to report adverse effects from participation in a GTA/MUTA activity (e.g., documentation and action steps to resolve the situation).	Comments: Consensus reached in Round 1		
1.1.11 Support GTAs/MUTAs who act in accordance with delineated program expectations if a complaint is made about them.	Mean (SD): 4.56 (0.51)<br>Comments: Consensus reached in Round 1	Consensus reached in Round 1	Consensus reached in Round 1
1.1.12 Manage client expectations of a GTA's/MUTA's possibilities and limitations.	Mean (SD): 4.38 (0.62)<br>Comments: Consensus reached in Round 1	Consensus reached in Round 1	Consensus reached in Round 1
1.1.13 Work with clients to clearly define the expected scope of GTA involvement in work assignments.	Mean (SD): 4.38 (1.02)<br>Comments: "Work with clients to clearly define the expected scope of GTA/MUTA involvement in work assignments." Based on comments to include MUTA.	Mean (SD): 4.6000 (0.63246)<br>Comments: Consensus reached in Round 2.	Consensus reached in Round 2
**New Additions**			
1.1.14	"Define and provide clear limitations to the specific skills that are permissible to instruct in a session (e.g. maximum number of exams, collection of samples, skills that are to be excluded from instruction)."	Mean (SD): 4.3333 (1.11270)<br>Comments: "Define and provide clear limitations regarding the scope of skills to be covered in an instructional session (e.g., maximum number of exams per day, exam techniques that must be included in each session, exam techniques that may be instructed but not practiced on a GTA/MUTA)." Revised for clarity.	Mean (SD): 4.67 (.49)<br>Comments: GTA/MUTA input on number of exams is important, but in order to reduce risk of personal compromise to health or safety, SP Educator should place reasonable limits. It can be difficult to make wise choices related to money. Consensus reached in Round 3
1.1.15	"Develop a policy or protocol for	Mean (SD): 4.6000 (0.50709)	Consensus reached in Round 2.

	injury reporting and medical management if an injury occurs."	Comments: Consensus reached in Round 2.	
1.1.16	"Reinforce techniques to reduce infection risk to self and others related to invasive examinations (e.g. proper handling of clean and contaminated equipment, hand hygiene, toileting)."	Mean (SD):4.4000 (0.91026) Comments: Consensus reached in Round 2.	Consensus reached in Round 2.
1.1.17	"Ensure acknowledgement of participants that they are aware of the nature of the instructional session prior to entering the room."	Mean (SD): 4.7333 (0.59362) Comments: Consensus reached in Round 2.	Consensus reached in Round 2.
**1.2 Confidentiality**			
1.2.1 Understand the specific principles of confidentiality that apply to all aspects of each simulation event.	Mean (SD): 4.31 (1.01) Comments: Consensus reached in Round 1. "Understand the specific principles of confidentiality that apply to all aspects of each instructional session." Based on comments reflecting inaccuracy of terminology.	Consensus reached in Round 1	Consensus reached in Round 1
1.2.2 Ensure that GTAs/MUTAs understand and maintain the principles of confidentiality related to specific simulation events.	Mean (SD): 4.31 (0.88) Comments: Consensus reached in Round 1. "Ensure that GTAs/MUTAs understand and maintain the principles of confidentiality related to specific instructional session." Based on comments reflecting inaccuracy of terminology.	Consensus reached in Round 1	Consensus reached in Round 1
1.2.3 Protect the privacy of the	Mean (SD): 4.44 (0.63)	Consensus reached in Round 1	Consensus reached in Round 1

personal information of all stakeholders, including that which may be revealed within a simulation activity.	Comments: Consensus reached in Round 1. “Protect the privacy of the personal information of all stakeholders, including that which may be revealed within an instructional session.” Based on comments reflecting inaccuracy of terminology.		
**New Additions**			
1.2.4	“Protect the privacy of any voice or video recording of GTA/MUTA instructional session”	Mean (SD): 4.4000 (1.24212) Comments: “Maintain instructor and student confidentiality by protecting the privacy of any voice or video recording related to a GTA/MUTA instructional session.” Revised for clarity.	Mean (SD): 4.87 (.35) Comments: Consensus reached in Round 3
**1.3 Respect**			
1.3.1 Respect GTA’s/MUTA’s self-identified boundaries (e.g., modesty, limits to physical touch, impact on person).	Mean (SD): 4.63 (0.50) Comments: Consensus reached in Round 1	Consensus reached in Round 1	Consensus reached in Round 1
1.3.2 Provide GTAs/MUTAs with adequate information so that they can make informed decisions about participation in work assignments.	Mean (SD): 4.63 (0.62) Comments: Consensus reached in Round 1	Consensus reached in Round 1	Consensus reached in Round 1
1.3.3 Ensure that GTAs/MUTAs understand if and how they are being compensated before accepting work (e.g., may include payment for training and work time, travel expenses, food vouchers, gift cards).	Mean (SD): 4.69 (0.48) Comments: Consensus reached in Round 1	Consensus reached in Round 1	Consensus reached in Round 1

	Not Applicable (1)	Somewhat Important (2)	Important (3)	Very Important (4)	Critically Important (5)
**Domain 2: Case Development** Comments: "Development of Instructional Materials"					
	**ROUND 1 RESULTS**		**ROUND 2 RESULTS**		**ROUND 3 RESULTS**
**2.1 Preparation**					
2.1.1 Ensure that cases align with measurable learning objectives.	Mean (SD): 3.19 (1.42)<br>Comments: "Ensure that training materials align with measurable learning objectives. "Based on comments reflecting inaccuracy of terminology.		Mean (SD): 3.21 (1.42)<br>Comments: "Ensure that instructional materials align with measurable learning objectives." Revised for clarity.		Mean (SD): 4.00 (1.07)<br>Comments: "Ensure that instructional materials align with measurable learning objectives."<br><br>Consensus reached in Round 3
2.1.2 Identify and engage relevant subject matter experts to assist in the creation of materials.	Mean (SD): 3.75 (1.13)<br>Comments: No additional comments		Mean (SD): 4.13 (.08)<br>Comments: Consensus reached in Round 2		Consensus reached in Round 2
2.1.3 Ensure that cases are based on authentic problems and respect the individuals represented in a case to avoid bias, or stereotyping marginalized populations.	Mean (SD): 3.00 (1.59)<br>Comments: "Ensure that instructional protocols are based on up-to-date clinical practice guidelines are based on authentic problems and respect the individuals represented in a case to avoid bias, or stereotyping marginalized populations." Diversity is reflected by the GTA/MUTA pool. Based on comments reflecting inaccuracy of terminology.		Mean (SD): 4.0000 (1.00000)<br>Comments: Consensus reached in Round 2. "Ensure that instructional protocols are based on up-to-date clinical practice guidelines, are based on authentic problems, and respect the individuals involved in or discussed during an instructional session to avoid bias, or stereotyping marginalized populations." Revised for clarity.		Consensus reached in Round 2
2.1.4 Ensure that case development process allows	Mean (SD): 2.94 (1.34)<br>Comments: "Ensure that		Mean (SD): 3.80 (1.08)<br>Comments: Consensus reached in		Consensus reached in Round 2

sufficient time to draft, review, and edit case materials prior to implementation.	development of training materials allows sufficient time to draft, review, and edit materials prior to implementation." Based on comments reflecting inaccuracy of terminology.	Round 2.	
2.1.5 Ensures that changes arising from dry-runs, or other piloting processes are addressed prior to implementation of the case.	Mean (SD): 3.13 (1.45) Comments: "Ensure that changes arising from dry-runs, or other piloting processes are addressed prior to implementation of the training materials." Based on comments reflecting inaccuracy of terminology.	Mean (SD): 3.53 (1.25) Comments: "Ensure that changes arising from piloting processes are addressed prior to implementation of the training materials." Revised for clarity.	Mean (SD): 4.07 (.60): Comments: "Ensure that changes arising from piloting processes are addressed prior to implementation of the training materials."<br><br>Consensus reached in Round 3
**2.2 Case Components. Ensure case components include the following when appropriate:**			
2.2.1 Clear goals and objectives that can be addressed.	Mean (SD): 3.87 (1.36) Comments: Consensus reached in Round 1	Consensus reached in Round 1	Consensus reached in Round 1
2.2.2 Goals and objectives that specify the intended level of learners.	Mean (SD): 3.69 (1.40) Comments: No additional comments	Mean (SD): 3.87 (.74) Comments: Consensus reached in Round 2.	Consensus reached in Round 2
2.2.3 Simulation design that meets the purpose.	Mean (SD): 3.75 (1.44) Comments: "Instructional design that meets the purpose." Based on comments reflecting inaccuracy of terminology.	Mean (SD): 4.20 (.94) Comments: Consensus reached in Round 2	Consensus reached in Round 2
2.2.4 Simulation design that is repeatable.	Mean (SD): 3.63 (1.36) Comments: "Instructional design that is repeatable." Based on comments reflecting inaccuracy of terminology.	Mean (SD): 4.20 (1.01) Comments: Consensus reached in Round 2	Consensus reached in Round 2
2.2.5 Information for GTAs/MUTAs (e.g., situation	Mean (SD): 2.87 (1.51) Comments: "Information for	Mean (SD): 4.47 (.74) Comments: Consensus reached in	Consensus reached in Round 2

and backstory, history, affect and demeanor, signs and symptoms to simulate, cues).	GTAs/MUTAs (e.g., description of physical examination techniques, cues)". Based on comments reflecting inaccuracy of terminology.	Round 2	
2.2.6 Training resources (e.g., props, moulage, videos, task trainers).	Mean (SD): 3.44 (1.15)<br>Comments: "Training resources (e.g. equipment, videos, task trainers)." Based on comments reflecting inaccuracy of terminology.	Mean (SD): 4.13 (.74)<br>Comments: Consensus reached in Round 2	Consensus reached in Round 2
2.2.7 Case-specific feedback or debriefing guidelines.	Mean (SD) 3.31 (1.35)<br>Comments: "Exam-specific feedback or debriefing guidelines." Based on comments reflecting inaccuracy of terminology.	Mean (SD): 4.00 (1.13)<br>Comments: "Guidelines for providing feedback to learners." Revised for clarity.	Mean (SD): 4.07 (.70):<br>Comments: "Guidelines for providing feedback to learners."<br><br>Consensus reached in Round 3
**2.2.8 Briefing instructions, time frames, instructions to learners.**			
2.2.9 Evaluation instruments and performance measures (e.g., checklists and rating scales, participant and facilitator evaluations).	Mean (SD): 3.50 (.89)<br>Comments: "Evaluation instruments and performance measures (e.g., checklists and rating scales, participant evaluations)." Based on comments reflecting inaccuracy of terminology.	Mean (SD): 3.73 (1.03)<br>Comments: "Evaluation instruments and performance measures<br>(e.g., participant evaluations) for learners<br>and/or GTAs/MUTAs." Revised for clarity regarding who is being evaluated.	Mean (SD): 3.73 (1.163):<br>Comments: one respondent disagreed that these components should be included in instructional materials<br><br>Suggested change: ""Evaluation instruments and performance measures<br>(e.g., participant evaluations) for learners and/or GTAs/MUTAs as applicable."<br><br>**Consensus not reached**
2.2.10 Training protocols for raters (GTA/MUTA or other).	Mean (SD): 3.56 (1.03)<br>Comments: "Training protocols for	Mean (SD): 3.33 (1.29)<br>Comments: "Training protocols for	Mean (SD): 3.53 (1.36):<br>Comments: one respondent

	GTA/MUTA rating of learner." Based on comments reflecting inaccuracy of terminology.	GTA/MUTA rating of learner."	indicated that this would only be applicable in a summative session, and another that often there is no rating of learners at all<br><br>Suggested change: "Training protocols for GTA/MUTA rating of learners as applicable."<br><br>**Consensus not reached**
2.2.11 Data for managing the documents and recruiting GTAs/MUTAs (e.g., author information, date of development, patient demographics, body type criteria).	Mean (SD): 3.33 (1.11)<br>Comments: No additional comments	Mean (SD): 3.29 (1.20)<br>Comments: "Data for managing the documents and recruiting GTAs/MUTAs (e.g., author information, date of development, bodily criteria)." Revised for clarity.	Mean (SD): 3.40 (1.06):<br>Comments: Respondents questioned necessity and clarity<br><br>Suggested change: "Data for managing the documentation related to the recruitment and retention of GTAs/MUTAs (e.g., author information, date of development, bodily criteria)."<br><br>**Consensus not reached**

	Not Applicable (1)	Somewhat Important (2)	Important (2)	Very Important (4)	Critically Important (5)
**Domain 3: GTA/MUTA Training**					
	**Round 1 Results**		**Round 2 Results**		**Round 3 Results**
**3.1 Preparation for Training**					
3.1.1 Review the purpose, objectives and outcomes, logistics, and case materials of the activity.	Mean (SD): 4.00 (.73)<br>Comments: Consensus reached in Round 1. "Review the purpose, objectives and outcomes, logistics, and instructional materials of the activity."		Consensus results in Round 1		Consensus results in Round 1
3.1.2 Address one's own knowledge gaps, if any.	Mean (SD): 4.25 (.68)<br>Comments: Consensus reached in Round 1. No additional comments		Consensus results in Round 1		Consensus results in Round 1
3.1.3 Create a training plan that is responsive to the context and format of each activity (e.g., group training for standardization, video review, practice with simulation equipment).	Mean (SD): 4.31 (.70)<br>Comments: Consensus reached in Round 1. "Create a training plan that is responsive to the context and format of each activity (e.g., group/peer training for standardization, video review, practice with simulation equipment)."		Consensus results in Round 1		Consensus results in Round 1
3.1.4 Gather training resources to supplement training.	Mean (SD): 3.94 (.85)<br>Comments: Consensus reached in Round 1. No comments		Consensus results in Round 1		Consensus results in Round 1
3.1.5 Gather administration documents and special instructions.	Mean (SD): 3.76 (.86)<br>Comments: No additional comments		Accidentally omitted from Round 2		Mean (SD): 3.67 (.82)<br>Comments: Consensus reached in Round 3
**3.2 Training for Role Portrayal**	***Replace with: "Training for Instructional Session"**				
3.2.1 Review with	Mean (SD): 4.44 (.51)		Consensus results in Round 1		Consensus results in Round 1

GTAs/MUTAs the key objectives, responsibilities, context (e.g., formative, summative, level of learner, placement in curriculum) and format (e.g., length of encounter, type of encounter) of each activity.	Comments: Consensus reached in Round 1. Variation in needs of cases versus instructional sessions.		
3.2.2 Engage GTAs/MUTAs in discussion and practice of role portrayal features (e.g. affect, signs and symptoms, behaviors).	Mean (SD): 3.20 (1.47)<br>Comments: "Engage GTAs/MUTAs in discussion and practice of instructional session features (e.g. techniques, behaviors, expectations, and guidance to provide)." Based on comments reflecting inaccuracy of terminology.	Mean (SD): 4.20 (.94)<br>Comments: Consensus reached in Round 2.	Consensus results in Round 2
3.2.3 Provide GTA/MUTAs with strategies to deal with unanticipated learner questions and behaviors.	Mean (SD): 4.19 (.75)<br>Comments: Consensus reached in Round 1. "Provide GTA/MUTAs with strategies to deal with unanticipated learner questions, behaviors, and/or actions."	Consensus results in Round 1	Consensus results in Round 1
3.2.4 Ensure consistency and accuracy of role portrayal of individual GTAs/MUTAs, and among groups of GTAs/MUTAs portraying the same role.	Mean (SD): 3.38 (1.41)<br>Comments: "Ensure consistency and accuracy of instructional session of individual GTAs/MUTAs, and among groups of GTAs/MUTAs with the same role." Based on comments reflecting inaccuracy of terminology.	Mean (SD): 4.47(.83)<br>Comments: Consensus reached in Round 2.	Consensus results in Round 2
3.2.5 Ensure GTA/MUTA readiness for the simulation activity through repeated practice and targeted feedback.	Mean (SD): 3.73 (1.39)<br>Comments: "Ensure GTA/MUTA readiness for the instructional session through repeated practice and	Mean (SD): 4.2.7 (1.10)<br>Comments: Consensus reached in Round 2.	Consensus results in Round 2

	targeted feedback." Based on comments reflecting inaccuracy of terminology.		
**New Additions**			
3.2.6	"Provide periodic refresher or re-calibration training, even if the instructional session does not change."	Mean (SD): 3.80 (1.01)<br>Comments: Consensus reached in Round 2.	Consensus results in Round 2
3.2.7	"Provide training on procedural skills (e.g. Pap collection) and equipment for training (e.g. task trainers) if used in educational sessions."	Mean (SD): 4.00 (1.13)<br>Comments: "Provide training on procedural skills (e.g. Pap collection) and equipment for training (e.g. task trainers) if utilized during instructional sessions." Revised for clarity	Mean (SD): 4.47 (.52)<br>Comments: Consensus reached in Round 3
3.2.8	"A screening examination provided by a healthcare provider may be used to demonstrate the sensations the GTA/MUTA may experience during an instructional session (e.g. demonstrate correct palpation of ovary) as well as to ensure that necessary reproductive organs are intact".	Mean (SD): 4.13 (.99)<br>Comments: "Provide an examination by a healthcare provider or other trained examiner to demonstrate the sensations the GTA/MUTA may experience during an instructional session (e.g. demonstrate correct palpation of ovary, prostate, etc)." Based on comments that this item was phrased awkwardly and incorporated components of both training and safety (addressed in Practice 1.1.3).	Mean (SD): 4.40 (1.12)<br>Comments: Support for inclusion of "other trained examiner". **Consensus not reached**
3.2.9	"Review current topics that apply to teaching session and/or case including consent, sexual violence, and communication."	Mean (SD): 3.47 (1.06)<br>Comments: "Review current topics that are applicable to and/or potentially impact instructional sessions (e.g., consent, sexual violence, communication)." Revised	Mean (SD): 3.80 (1.01)<br>Comments: Consensus reached in Round 3

		for clarity.	
3.2.10	"Reinforce techniques to reduce infection risk to self and others related to invasive examinations (e.g. proper handling of clean and contaminated equipment, hand hygiene, toileting)"	Mean (SD): 4.33 (.72)<br>Comments: Repetitive from Practice 1.1.16. Based on comments that infection control is primarily a safety concern, this Practice will be removed from Domain 3 and remain in Domain 1.	Removed
3.2.11	"Reinforce techniques to reduce risk of physical and psychological harm to GTA/MUTA and learner (e.g. hand hygiene, appropriate touch, consent, terminology, communication techniques)."	Mean (SD): 4.27 (.70)<br>Comments: Consensus reached in Round 2. "Educate regarding and reinforce techniques to reduce risk of physical and psychological harm to GTA/MUTA and learner (e.g. appropriate touch, appropriate pressure, consent, terminology, communication techniques)." Hand hygiene removed from list of examples due to repetition from 1.1.16.	Consensus results in Round 2
3.2.12	"Provide GTAs/MUTAs with the ability to demonstrate proficiency in examination maneuvers that they will be instructing."	Mean (SD): 4.27 (1.16)<br>Comments: Consensus reached in Round 2. "Provide GTAs/MUTAs with the opportunity to demonstrate proficiency in examination maneuvers that they will be instructing."<br>Revised for clarity.	Consensus results in Round 2
3.2.13	"Ensure pre-session resources are provided to learners to prepare them for the teaching sessions (ex. institution-prepared materials, textbook reading assignments related to gynecological and male urological	Mean (SD): 4.07 (1.10)<br>Comments: Consensus reached in Round 2. "Ensure pre-session resources are provided to learners to prepare them for the teaching sessions (e.g.,. institution-prepared materials,	Consensus results in Round 2

	and prostate exams, etc.)."	textbook reading assignments related to gynecological and urological and prostate exams, etc.)." Revised to removed gendered terminology.	
**3.3 Training for Feedback**			
3.3.1 Review with GTAs/MUTAs the fundamental principles of feedback as they related to the planned activity.	Mean (SD): 3.94 (.77)<br>Comments: "Review with GTAs/MUTAs the fundamental principles of feedback as an instructional methodology to be applied to the planned activity." Real-time feedback supports learning.	Mean (SD): 4.07 (.96)<br>Comments: Consensus reached in Round 2.	Consensus results in Round 2
3.3.2 Inform GTAs/MUTAs of the feedback objectives and level of the learners with whom they will be learning.	Mean (SD): 3.88 (1.09)<br>Comments: No additional comments.	Mean (SD): 3.93 (1.10)<br>Comments: Consensus reached in Round 2. "Inform GTAs/MUTAs of the feedback objectives and level of the learners with whom they will be working." Revised for clarity.	Consensus results in Round 2
3.3.3 Inform GTAs/MUTAs of the feedback logistics and setting (e.g., one-on-one feedback with learner, small group feedback, simulation debrief).	Mean (SD): 3.69 (1.25)<br>Comments: "Inform GTAs/MUTAs of the feedback logistics and setting (e.g., one-on-one feedback with learner, small group feedback)."	Mean (SD): 3.80 (1.08)<br>Comments: "Inform GTAs/MUTAs of the feedback logistics and setting (e.g., individual feedback with learner, small group feedback, written feedback, intended structure)." Revised for clarity of setting and logistics.	Mean (SD): 3.93 (.88)<br>Comments: Consensus reached in Round 3
3.3.4 Train GTAs/MUTAs to use their observations, responses, and knowledge to provide feedback on observable, modifiable behaviors in learners.	Mean (SD): 4.13 (1.02)<br>Comments: "Train GTAs/MUTAs to use their observations, bodily sensations, and knowledge to provide feedback on observable, modifiable behaviors in learners."	Mean (SD): 4.53 (.92)<br>Comments: Consensus reached in Round 2.	Consensus results in Round 2
3.3.5 Ensure GTA/MUTA	Mean (SD): 4.00 (1.21)	Mean (SD): 4.01 (1.49)	Mean (SD): 4.27 (1.03)

readiness through repeated practice and targeted feedback.	Comments: No additional comments	Comments: There is some potential redundancy with Practice 3.2.12 and 3.2.4.	Comments: Consensus reached in Round 3
**New Additions**			
3.3.6	"Train GTAs/MUTAs to utilize communication techniques that optimize learning outcomes during instructional sessions (e.g. correction of technique, use of inquiry, avoidance of leading questions, etc)"	Mean (SD): 3.73 (1.22)<br>Comments: Repetitive from Practice 3.3.1. This Practice will be removed and clarification will be provided on effective provision of feedback as an instructional methodology during the introduction to this Domain.	Removed
3.3.7	"Train GTAs/MUTAs to recognize and respond to a learner that is having a negative experience during the instructional session (e.g. history to violence, discomfort with anatomy, etc), including provision of resources."	Mean (SD): 4.13 (1.25)<br>Comments: "Train GTAs/MUTAs to recognize and respond to a learner that is having a negative experience during the instructional session (e.g. history of violence, discomfort with anatomy, etc), with the intention of ensuring a safe, nontraumatic learning environment." Revised for clarity.	Mean (SD): 4.40 (.63))<br>Comments: In alignment with Domain 1. Consensus reached in Round 3
3.3.8	"Review methods of promoting instructional effectiveness through real-time feedback"	Mean (SD): 3.47 (1.41)<br>Comments: No additional comments	Mean (SD): 3.73 (1.03)<br>Comments: **Consensus not reached**
3.3.9	"Provide examples of challenging scenarios that may arise during a teaching session or case (e.g. learner without exposure to another's genital anatomy, learner with personal history of sexual violence, GTA/MUTA physical or psychological trigger)."	Mean (SD): 3.67 (1.05)<br>Comments: Intention is repetitive of Practice 3.3.7 and 4.3.7, so this Practice will be removed.	Removed
**3.4 Training for Completion of Assessment Instruments**			
3.4.1 Ensure that GTAs/MUTAs	Mean (SD): 3.63 (1.31)	Mean (SD): 3.60 (1.60)	Mean (SD): 3.73 (1.16)

understand the nature, context, and objectives of the assessment.	Comments: Useful only in programs that use GTAs/MUTAs for assessment of skills. Most programs use GTAs/MUTAs for instruction, not for assessment of skills.	Comments: Useful only in programs that use GTAs/MUTAs for assessment of skills. Most programs use GTAs/MUTAs for instruction, not for assessment of skills.	Comments: Critical for programs that do assess. **Consensus not reached**
3.4.2 Ensure that GTAs/MUTAs understand the format of the assessment instrument.	Mean (SD): 3.56 (1.41) Comments: Useful only in programs that use GTAs/MUTAs for assessment of skills. Most programs use GTAs/MUTAs for instruction, not for assessment of skills.	Mean (SD): 3.60 (1.55) Comments: Most programs use GTAs/MUTAs for instruction, not for assessment of skills.	Mean (SD): 3.60 (1.30) Comments: Critical for programs that do assess. **Consensus not reached**
3.4.3 Ensure that GTAs/MUTAs are able to complete assessment instruments in the time allotted.	Mean (SD): 3.31 (1.40) Comments: Useful only in programs that use GTAs/MUTAs for assessment of skills. Most programs use GTAs/MUTAs for instruction, not for assessment of skills.	Mean (SD): 3.40 (1.50) Comments: Most programs use GTAs/MUTAs for instruction, not for assessment of skills.	Mean (SD): 3.53 (1.36) Comments: Critical for programs that do assess. **Consensus not reached**
3.4.4 Provide GTAs/MUTAs with practice completing assessment instruments with a variety of learner behaviors.	Mean (SD): 2.94 (1.34) Comments: Useful only in programs that use GTAs/MUTAs for assessment of skills. Most programs use GTAs/MUTAs for instruction, not for assessment of skills.	Mean (SD): 3.00 (1.65) Comments: Most programs use GTAs/MUTAs for instruction, not for assessment of skills.	Mean (SD): 3.07 (1.22) Comments: Critical for programs that do assess. **Consensus not reached**
3.4.5 Ensure that GTAs/MUTAs understand both the principle and receptive experiences of any physical exam maneuvers they will be assessing.	Mean (SD): 3.63 (1.50) Comments: Useful only in programs that use GTAs/MUTAs for assessment of skills. Most programs use GTAs/MUTAs for instruction, not for assessment of skills.	Mean (SD): 3.67 (1.63) Comments: Most programs use GTAs/MUTAs for instruction, not for assessment of skills.	Mean (SD): 3.60 (1.40) Comments: Critical for programs that do assess. Concern regarding "receptive experience given the context of the exam **Consensus not reached**
3.4.6 In formative assessment ensure consistent and accurate completion of an assessment	Mean (SD): 3.56 (1.41) Comments: Useful only in programs that use GTAs/MUTAs for	Mean (SD): 3.20 (1.52) Comments: Useful only in programs that use GTAs/MUTAs for	Mean (SD): 3.73 (1.17) Comments: Critical for programs that do assess. **Consensus not reached**

instrument within individual GTAs/MUTAs, and among groups of GTAs/MUTAs performing the same task.	assessment of skills. Most programs use GTAs/MUTAs for instruction, not for assessment of skills.	assessment of skills. Most programs use GTAs/MUTAs for instruction, not for assessment of skills.	
3.4.7 In high stakes assessment, verify inter-rater reliability, in which a learner would achieve the same score when rated by different GTAs/MUTAs.	Mean (SD): 3.25 (1.73)<br>Comments: Useful only in programs that use GTAs/MUTAs for high-stakes assessment. Even among programs that use GTAs/MUTAs for assessment, it is still typically not high-stakes.	Mean (SD): 2.73 (1.83)<br>Comments: Most programs use GTAs/MUTAs for instruction, not for assessment of skills.	Mean (SD): 3.73 (1.53)<br>Comments: Critical for programs that do assess. **Consensus not reached**
3.4.8 In high stakes assessment, verify intra-rater reliability, in which GTAs/MUTAs would assign the same score to an identical performance at different points in time.	Mean (SD): 3.31 (1.78)<br>Comments: Useful only in programs that use GTAs/MUTAs for high-stakes assessment. Even among programs that use GTAs/MUTAs for assessment, it is still typically not high-stakes.	Mean (SD): 2.73 (1.83)<br>Comments: Most programs use GTAs/MUTAs for instruction, not for assessment of skills.	Mean (SD): 3.67 (1.54)<br>Comments: Critical for programs that do assess. Need clarity regarding "time". **Consensus not reached**
**3.5 Reflection on the Training Process**			
3.5.1 Reflect on one's own training practices for future improvement (e.g., evaluation forms, debriefing, video review.	Mean (SD): 4.19 (.75)<br>Comments: Consensus reached in Round 1. No additional comments	Consensus results in Round 1	Consensus reached in Round 1

	Not Applicable (1)	Somewhat Important (2)	Important (3)	Very Important (4)	Critically Important (5)
**Domain 4: Program Management**					
	**ROUND 1 RESULTS**		**ROUND 2 RESULTS**		**ROUND 3 RESULTS**
**4.1 Purpose**					
4.1.1 Articulate a mission statement for the program.	Mean (SD): 3.88 (1.09)<br>Comments: Consensus reached in Round 1		Consensus reached in Round 1		Consensus reached in Round 1
4.1.2 Develop program goals.	Mean (SD): 4.06 (.77)<br>Comments: Consensus reached in Round 1		Consensus reached in Round 1		Consensus reached in Round 1
4.1.3 Identify measurable objectives for each goal (where applicable).	Mean (SD): 3.94 (.93)<br>Comments: Consensus reached in Round 1		Consensus reached in Round 1		Consensus reached in Round 1
**4.2 Expertise**					
4.2.1 Possess depth of knowledge in GTA/MUTA methodology.	Mean (SD): 4.44 (.73)<br>Comments: Consensus reached in Round 1		Consensus reached in Round 1		Consensus reached in Round 1
4.2.2 Advocate for the integration of GTA/MUTA methodology into the curriculum where appropriate.	Mean (SD): 4.00 (.73)<br>Comments: Consensus reached in Round 1		Consensus reached in Round 1		Consensus reached in Round 1
4.2.3 Identify when GTAs/MUTAs should be incorporated into a simulation activity.	Mean (SD) 3.56 (1.15)<br>Comments: "Identify when GTAs/MUTAs should be incorporated into an instructional session". Based on comments reflecting inaccuracy of terminology.		Mean (SD): 3.47 (1.30)<br>Comments: No additional comments		Mean (SD): 4.07 (.80)<br>Comments: Consensus reached in Round 3
4.2.4 Collaborate with subject	Mean (SD): 4.38 (.89)		Consensus reached in Round 1		Consensus reached in Round 1

matter experts to design GTA/MUTA cases, training, and assessment materials.	Comments: Consensus reached in Round 1. "Collaborate with subject matter experts to design GTA/MUTA instructional sessions and materials"		
4.2.5 Train GTAs/MUTAs according to scenario or project parameters.	Mean (SD): 4.15 (.81)<br>Comments: Consensus reached in Round 1. "Train GTAs/MUTAs according to the parameters of instructional session."	Consensus reached in Round 1	Consensus reached in Round 1
**4.3 Policies and Procedures**			
4.3.1 Develop and document policies to guide program activities.	Mean (SD): 4.25 (.77)<br>Comments: Consensus reached in Round 1	Consensus reached in Round 1	Consensus reached in Round 1
4.3.2 Develop and document policies that take into consideration disability access and inclusion.	Mean (SD): 3.50 (1.32)<br>Comments: Need clarification for "whose disability"?	Mean (SD): 3.47 (1.126)<br>Comments: This Practice is in alignment with 4.3.6, which has been modified to incorporate ability. This Practice will be omitted.	Removed
4.3.3 Develop and document business processes and procedures, including but not limited to creating financial management, business, and strategic plans.	Mean (SD): 3.62 (1.54)<br>Comments: no additional comment	Mean (SD): 3.13 (1.552)<br>Comments: This does not apply specifically to GTAs/MUTAs, but to the broader organization they exist within - just the same as all other items within Domain 4.	Mean (SD): 3.47 (.99)<br>Comments: **Consensus not reached**
4.3.4 Ensure policies and procedures are kept current and accessible.	Mean (SD): 4.38 (.72)<br>Comments: Consensus reached in Round 1	Consensus reached in Round 1	Consensus reached in Round 1
4.3.5 Distribute policies and procedures to relevant stakeholders.	Mean (SD): 4.25 (.86)<br>Comments: Consensus reached in Round 1	Consensus reached in Round 1	Consensus reached in Round 1
**New Additions**			

4.3.6	"Develop and document policies that protect groups from discrimination based on sex, race, religion, color, national origin, age, sexual orientation, and/or gender presentation"	Mean (SD): 4.40 (0.63)<br>Comments: " Develop and document policies that protect groups from discrimination based on ability, age, race, ethnicity, skin color, national origin, religion, sex, sexual orientation, gender identity, and/or gender presentation." Revised to incorporate intentions of Practices 4.3.2 and 4.3.8.	Mean (SD): 3.87 (.99)<br>Comments: Consensus reached in Round 3
4.3.7	"Develop and document policies for termination of teaching session and/or case related to GTA/MUTA or learner concern."	Mean (SD): 4.00 (1.20)<br>Comments: Consensus reached in Round 2	Consensus reached in Round 2
4.3.8	"Develop and document terminology and policies that reduce gender bias regarding GTAs/MUTAs (e.g. remove male from MUTA)."	Mean (SD): 2.87 (1.69)<br>Comments: This Practice is in alignment with 4.3.6, which has been modified to incorporate sex, gender identity, and gender presentation. This Practice will be omitted.	Removed
4.3.9	"Develop and document policies regarding various bodily processes that may impact and/or occur during teaching sessions and/or scenarios (e.g. discharge, vaginal bleeding, erection, passing gas or stool, infection)."	Mean (SD): 4.07 (1.33)<br>Comments: Consensus reached in Round 2	Consensus reached in Round 2
**New Item**			
4.3.10	-	"Develop a policy or protocol for instruction of procedural skills during GTA/MUTA sessions (e.g., collection of samples)." Created in response to feedback for 1.1.14.	Mean (SD): 4.20 (1.15)<br>Comments: Consensus reached in Round 3
**4.4 Records Management**			
4.4.1 Collaborate with subject	Mean (SD): 3.37 (1.09)	Mean (SD): 3.13 (1.60)	Mean (SD): 3.33 (1.23)

matter experts to develop a system for reporting learner performance to stakeholders (e.g., learners, curriculum developers, faculty, administration).	Comments: no additional comment	Comments: Some concern about repetition with Domain 2, but there collaboration was for preparation for the instructional session whereas this is addressing the results of the session.	Comments: **Consensus not reached**
4.4.2 Ensure that policies are in place for case sharing and archiving.	Mean (SD): 2.62 (1.20)<br>Comments: "Ensure that policies are in place for sharing and archiving the materials of instructional sessions". Based on comments reflecting inaccuracy of terminology.	Mean (SD): 3.87 (1.30)<br>Consensus reached in Round 2	Consensus reached in Round 2
4.4.3 Develop and document methods for securely storing, archiving, and destroying confidential data (e.g., SP records, learner data, video data, consent forms, release forms).	Mean (SD): 3.81 (1.22)<br>Comments: "Develop and document methods for securely storing, archiving, and destroying confidential data (e.g., GTA/MUTA records, learner data, video data, consent forms, release forms)."	Mean (SD): 4.07 (1.39)<br>Consensus reached in Round 2	Consensus reached in Round 2
**4.5 Team Management**			
4.5.1 Consult with legal, financial, and human resources experts to ensure that status of SPs (e.g., employee, independent contractor, volunteer) and compensation structure (if applicable) comply with institutional requirements.	Mean (SD): 4.25 (.93)<br>Comments: Consensus reached in Round 1. "Consult with legal, financial, and human resources experts to ensure that status of GTAs/MUTAs (e.g., employee, independent contractor, volunteer) and compensation structure (if applicable) comply with institutional requirements."	Consensus reached in Round 1	Consensus reached in Round 1
4.5.2 Develop processes to identify, screen, interview, select, debrief, and maintain	Mean (SD): 4.63 (.62)<br>Comments: Consensus reached in Round 1	Consensus reached in Round 1	Consensus reached in Round 1

GTAs/MUTAs and staff.			
4.5.3 Recruit and maintain a cohort of GTAs/MUTAs that reflects the diversity of the people they represent in simulation activities.	Mean (SD): 3.81 (1.17)<br>Comments: "Recruit and maintain a cohort of GTAs/MUTAs that reflects the diversity of the people they represent in instructional sessions."	Mean (SD): 3.87 (0.83)<br>Comments: Consensus reached in Round 2.<br>"Recruit and maintain a cohort of GTAs/MUTAs that is inclusive and diverse." Revised for clarity and to reflect that GTAs/MUTAs do not represent others'.	Consensus reached in Round 2
4.5.4 Establish policies and procedures for the psychological, physical, and environmental safety of GTAs/MUTAs, learners, staff, and faculty.	Mean (SD): 4.63 (.62)<br>Comments: Consensus reached in Round 1	Consensus reached in Round 1	Consensus reached in Round 1
4.5.5 Advocate for ongoing professional development opportunities for all staff, including GTAs/MUTAs.	Mean (SD): 4.00 (1.03)<br>Comments: Consensus reached in Round 1	Consensus reached in Round 1	Consensus reached in Round 1
**4.6 Quality Management**			
4.6.1 Gather data regularly to assess the alignment of program activities with legislated, institutional, and program policies and procedures.	Mean (SD): 3.75 (1.18)<br>Comments: no additional comment	Mean (SD): 3.60 (1.18)<br>Comments: no additional comments	Mean (SD): 3.40 (.99)<br>Comments: **Consensus not reached**
4.6.2 Gather feedback regularly from GTAs/MUTAs, learners, faculty, and other users regarding the quality of services provided by the program.	Mean (SD) 4.38 (.72)<br>Comments: Consensus reached in Round 1	Consensus reached in Round 1	Consensus reached in Round 1
4.6.3 Analyze data and other feedback in a timely manner.	Mean (SD): 3.9 (.77)<br>Comments: Consensus reached in Round 1	Consensus reached in Round 1	Consensus reached in Round 1

4.6.4 Implement changes for continuous improvement.	Mean (SD): 4.06 (.77)<br>Comments: Consensus reached in Round 1	Consensus reached in Round 1	Consensus reached in Round 1
4.6.5 Inform stakeholders of changes made based on their feedback.	Mean (SD): 3.94 (.77)<br>Comments: Consensus reached in Round 1	Consensus reached in Round 1	Consensus reached in Round 1

	Not Applicable (1)	Somewhat Important (2)	Important (3)	Very Important (4)	Critically Important (5)
**Domain 5: Professional Development**					
	**ROUND 1 RESULTS**		**ROUND 2 RESULTS**		**ROUND 3 RESULTS**
**5.1 Career Development**					
5.1.1 Develop and promote expertise in knowledge, skills, and attitudes related to GTA/MUTA-based simulation.	Mean (SD): 3.75 (1.13)<br>Comments: "Develop and promote expertise in knowledge, skills, and attitudes related to GTA/MUTA-based instructional sessions"<br>Based on comments reflecting inaccuracy of terminology.		Mean (SD): 3.73 (0.70)<br>Comments: no additional comments		Mean (SD): 3.67 (1.11)<br>Comments: **Consensus not reached**
5.1.2 Develop and promote expertise in theories, principles, and processes of education and assessment relevant to the context of one's practice (e.g., medical education, nursing education, legal, and law enforcement training).	Mean (SD): 3.37 (.89)<br>Comments: no additional comments		Mean (SD): 3.21 (1.37)<br>Comments: no additional comments		Mean (SD): 3.53 (.99)<br>Comments: GTA/MUTAs are unlikely to work in legal or law enforcement training capacities. Removing those examples would provide clarity. **Consensus not reached**
5.1.3 Maintain membership in professional simulation societies (e.g., ASPE, ASPiH, INACSL, SESAM, SSH).	Mean (SD): 3.37 (1.20)<br>Comments: no additional comments		Mean (SD): 3.00 (1.31)<br>Comments: no additional comments		Mean (SD): 3.33 (1.23)<br>Comments: **Consensus not reached**
5.1.4 Engage in educational opportunities (e.g., professional conferences, courses, degree programs,	Mean (SD): 3.43 (.73)<br>Comments: no additional comments		Mean (SD): 3.27 (1.39)<br>Comments: no additional comments		Mean (SD): 3.47 (1.30)<br>Comments: **Consensus not reached**

certifications).			
5.1.5 Develop personal management skills (e.g., time management, wellness strategies, career planning).	Mean (SD): 3.43 (1.03)<br>Comments: no additional comments	Mean (SD): 3.00 (1.37)<br>Comments: no additional comments	Mean (SD): 3.27 (1.10)<br>Comments: **Consensus not reached**
5.1.6 Seek out opportunities for career mentoring.	Mean (SD): 3.00 (1.10)<br>Comments: no additional comments	Mean (SD): 2.67 (1.18)<br>Comments: no additional comments	Mean (SD): 2.93 (1.16)<br>Comments: **Consensus not reached**
**5.2 Scholarship**			
5.2.1 Develop an understanding of the range of opportunities for scholarship in GTA/MUTA methodology.	Mean (SD): 3.00 (1.26)<br>Comments: no additional comments	Mean (SD): 2.67 (1.54)<br>Comments: no additional comments	Mean (SD): 3.07 (1.22)<br>Comments: **Consensus not reached**
5.2.2 Identify and/or develop new contexts for SP methodology.	Mean (SD): 3.00 (1.10)<br>Comments: "Identify and/or develop new contexts for GTA/MUTA methodology" Based on comments reflecting inaccuracy of terminology.	Mean (SD): 2.53 (1.41)<br>Comments: This responsibility may be shared with faculty.	Mean (SD): 3.00 (1.13)<br>Comments: **Consensus not reached**
5.2.3 Contribute to the evolution of best practices through innovation, research, and dissemination of emerging methods in various venues (e.g., publications, presentations).	Mean (SD): 3.62 (.72)<br>Comments: no additional comments	Mean (SD): 3.20 (1.42)<br>Comments: no additional comments	Mean (SD): 3.20 (1.08)<br>Comments: Important, but aspirational. **Consensus not reached**
**5.3 Leadership**			
5.3.1 Promote understanding and development of GTA/MUTA methodology locally, nationally, and internationally.	Mean (SD): 3.75 (1.00)<br>Comments: no additional comments	Mean (SD): 3.33 (1.29)<br>Comments: no additional comments	Mean (SD): 3.47 (1.06)<br>Comments: **Consensus not reached**

5.3.2 Mentor and support GTAs/MUTAs and other GTA/MUTA educators within one's institution and within the community of practice.	Mean (SD): 3.68 (1.01)<br>Comments: no additional comments	Mean (SD): 3.8667 (1.18723)<br>Consensus reached in Round 2	Consensus reached in Round 2
5.3.3 Seek out and advocate for growth of leadership skills (e.g., collaboration, team building, change management, interpersonal effectiveness, conflict resolution).	Mean (SD): 3.43 (1.03)<br>Comments: no additional comments	Mean (SD): 3.27 (1.49)<br>Comments: no additional comments	Mean (SD): 3.07 (1.28)<br>Comments: **Consensus not reached**
